# Single-Atom Ru on N-Heterocyclic Carbene-Functionalized Hypercrosslinked Polymers for N-Formylation of Amines with CO_2_ and H_2_

**DOI:** 10.3390/molecules31173054

**Published:** 2026-08-31

**Authors:** Lulu Dang, Yinfang Gao, Yali Wan, Qingsong Li, Yizhu Lei

**Affiliations:** 1 Guizhou Provincial Key Laboratory of Green Catalysis and Materials for Resource Conversion, School of Chemistry and Materials Engineering, Liupanshui Normal University, Liupanshui 553004, China; 18535884136@163.com (L.D.); 15365116330@163.com (Y.G.); 2 College of Environmental and Chemical Engineering, Dalian University, Dalian 116622, China; 3 School of Chemical Engineering, Guizhou Institute of Technology, Guiyang 550025, China; 4 Guizhou Laboratory of Energy Intelligent Development and Efficient Utilization, Guizhou Research Institute of Coal Mine Design Co., Ltd., Guiyang 550025, China; liqingsong@126.com

**Keywords:** CO_2_ utilization, single-atom catalyst, Ru, N-formylation, porous polymer

## Abstract

Catalytic transformation of carbon dioxide (CO_2_) into high-value-added chemicals represents a promising approach for CO_2_ emission reduction and utilization. Herein, single-atom ruthenium (Ru) supported on N-heterocyclic carbene (NHC)-functionalized hypercrosslinked polymers were fabricated and applied as recyclable catalysts for the N-formylation of amines using CO_2_ and H_2_ as feedstocks. Catalytic evaluations demonstrated that the chemical structure of the NHC ligand had a significant influence on catalytic activity. Specifically, P(PhPy-TPB)-Ru, functionalized with a single pyridine moiety on the NHC ligand, exhibited the optimal catalytic performance, even slightly outperforming its homogeneous counterpart. Furthermore, mechanistic studies indicated that both the base and solvent played pivotal roles in modulating the reaction activity. In particular, the use of methanol as the solvent in the presence of a base significantly enhanced reaction efficiency, which was attributed to the in situ generation of methyl formate as a key intermediate. Notably, the developed catalytic system possessed excellent catalytic efficiency, a broad substrate scope, superior stability, and easy recyclability, allowing the catalyst to be reused for up to eight consecutive cycles without significant loss of catalytic activity.

## 1. Introduction

The growing accumulation of atmospheric carbon dioxide (CO_2_) has precipitated severe global environmental challenges, making the development of efficient CO_2_ capture and conversion strategies a critical research priority in chemistry and environmental sciences [1,2,3]. Catalytic conversion of CO_2_ into high-value fine chemicals not only reduces greenhouse gas emissions but also facilitates the valorization of this abundant, non-toxic C1 feedstock [4,5], which conforms to the fundamental principles of green and sustainable chemistry. Among various CO_2_ conversion reactions, the N-formylation of amines to formamides is of particular industrial and academic significance, as formamide derivatives serve as key intermediates in the synthesis of pharmaceuticals, agrochemicals, fine chemicals and functional materials [6,7]. Traditional N-formylation processes typically employ toxic C1 sources such as CO [8], formic acid [9] and formaldehyde [10]. In comparison, the direct N-formylation of amines employing CO_2_ and molecular hydrogen (H_2_) as reactants constitutes a more environmentally benign strategy [6,11,12,13,14], which has consequently garnered extensive research interest in recent years.

Heterogeneous catalysis has established itself as an efficient strategy for CO_2_-mediated N-formylation reactions, owing to the easy separability and recyclable nature of the catalysts [15,16,17,18,19,20,21]. Porous organic polymers (POPs), meanwhile, have emerged as ideal catalyst supports by virtue of their high specific surface area, tunable chemical structures, excellent thermal stability, and designable functional groups [22,23,24]. Phosphorus-containing POPs have been extensively studied for this transformation and have demonstrated promising catalytic performance, as phosphine ligands are capable of efficiently coordinating with metal active sites and regulating their electronic properties [25,26,27,28,29]. Nevertheless, phosphine ligands possess inherent limitations related to poor stability, including facile oxidation under ambient or reaction conditions [30,31]; this phenomenon results in the deactivation of metal active sites and compromises the long-term recyclability of phosphorus-functionalized catalysts. N-heterocyclic carbenes (NHCs), as a class of nitrogen-containing ligands, exhibit superior chemical and thermal stability [32,33,34]. Their strong σ-donating capacity and tunable steric structure facilitate efficient coordination with metal centers to form stable catalytic sites, thereby positioning them as a promising alternative to phosphine ligands for the fabrication of robust heterogeneous catalysts.

Hypercrosslinked polymers (HCPs), a subclass of POPs, possess hierarchical porous structures, high specific surface areas and facile functionalization, which can not only disperse metal active sites uniformly but also facilitate the mass transport of reactants and products during catalysis [35,36]. Single-atom metal catalysts (SACs) maximize atomic utilization efficiency and exhibit unique catalytic activity and selectivity owing to their isolated atomic active sites [37], and the combination of NHC-functionalized HCPs and single-atom metals is expected to fabricate high-performance heterogeneous catalysts for CO_2_ conversion reactions [38,39,40,41,42]. Nevertheless, systematic investigations on the regulation of NHC ligand structures (e.g., introducing pyridine moieties) to optimize the CO_2_ adsorption, activation ability and catalytic performance of single-atom metal catalysts for the N-formylation of amines with CO_2_ and H_2_ remain scarce [40].

In this work, we designed and synthesized a series of single-atom ruthenium (Ru) catalysts supported on pyridine-modified NHC-functionalized hypercrosslinked polymers via a Friedel–Crafts alkylation and subsequent metal complex grafting strategy. The effect of the number of pyridine moieties on the NHC ligand on the physicochemical properties and catalytic performance of the catalysts was systematically investigated for the CO_2_/H_2_-mediated N-formylation of amines. The reaction conditions including solvent, pressure, base dosage and temperature were optimized, and the recyclability and substrate scope of the optimal catalyst were evaluated in detail. Furthermore, the reaction mechanism was elucidated through control experiments, confirming methyl formate as the key intermediate in the reaction system with methanol as the solvent. This study provides a feasible strategy for the rational design of stable, efficient and recyclable nitrogen-functionalized POP-supported single-atom metal catalysts for CO_2_ valorization reactions.

## 2. Results and Discussion

### 2.1. Catalyst Design and Characterization

**Scheme 1:**
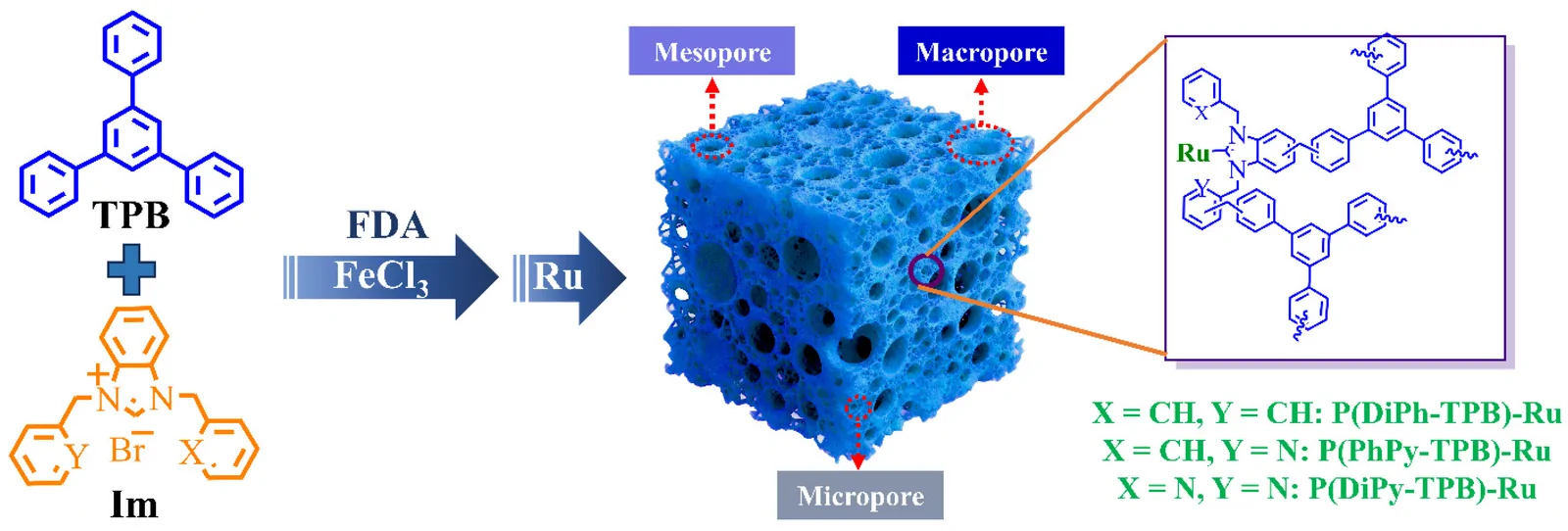
Schematic representation for the synthesis of single-atom Ru on N-heterocyclic carbene-functionalized hypercrosslinked polymers.

Scheme 1 illustrates the synthesis route of single-atom ruthenium (Ru) supported on N-heterocyclic carbene (NHC)-functionalized hypercrosslinked polymers. As depicted, hypercrosslinked polymer precursors bearing diverse NHC structures were fabricated via Friedel–Crafts alkylation of NHC precursors and triphenylbenzene, using formaldehyde dimethyl acetal as the crosslinker and anhydrous FeCl_3_ as the catalyst. Considering that pyridine moieties can significantly influence CO_2_ adsorption, activation capability, and catalytic performance of the resulting catalysts, NHC precursors with different numbers of pyridine functional groups were deliberately used to regulate the catalytic behavior. Subsequently, Ru complexes were immobilized onto the NHC-functionalized POPs by treating [Ru(CO)_3_Cl_2_]_2_ in methanol with triethylamine as the base.

**Figure 1:**
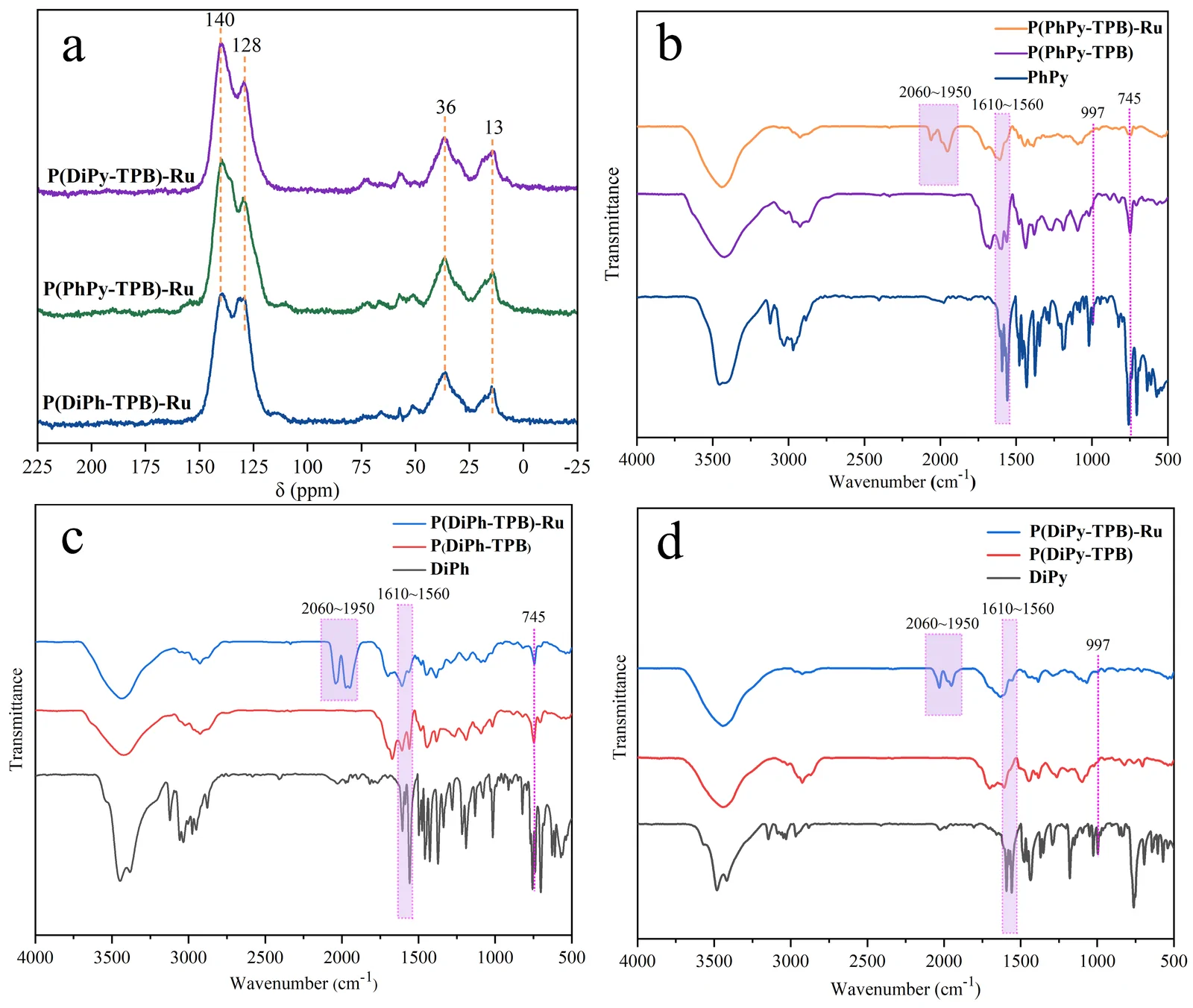
(**a**) Solid-state ^13^C CP-MAS NMR spectra; (**b**–**d**) FTIR spectra.

Solid-state ^13^C CP-MAS NMR spectra (Figure 1a) of the as-synthesized catalysts exhibited two resonance signals at approximately 140 ppm and 128 ppm, corresponding to quaternary aromatic carbons and protonated aromatic carbons, respectively [43]. Meanwhile, the aliphatic carbon signals detected at roughly 36 ppm were attributed to the methylene carbon of the crosslinker (connecting two aromatic rings), which was generated via the Friedel–Crafts reaction [44]. As shown in Figure 1b–d, with respect to the FT-IR spectra of P(PhPy-TPB)-Ru, P(DiPh-TPB)-Ru, and P(DiPy-TPB)-Ru, all samples exhibited characteristic absorption bands in the range of 2060–1950 cm^−1^, which were assigned to the ν(CO) stretching vibrations of coordinated carbonyl ligands, confirming the successful immobilization of Ru species onto the polymer frameworks [27]. For all samples, the bands at 1610–1561 cm^−1^ were attributed to the stretching vibrations of C = N and C = C bonds [45]. The characteristic peaks in the 1250–950 cm^−1^ and 900–600 cm^−1^ regions were assigned to the out-of-plane bending and in-plane bending vibrations of aromatic C-H bonds, respectively [45]. A characteristic band at 997 cm^−1^ was observed for P(PhPy TPB)Ru, P(DiPy TPB)Ru, their precursors and corresponding monomers (Figure 1b,d), which is assigned to the ring-breathing vibration of the pyridine rings in the DiPy and PhPy moieties. Meanwhile, the characteristic absorption at 745 cm^−1^ appeared in P(PhPy TPB)Ru, P(DiPh TPB)Ru, their precursors and monomers (Figure 1b,c), corresponding to the out-of-plane C–H bending vibration of benzene rings in the DiPh and PhPy units. These results confirm the successful incorporation of PhPy, DiPy and DiPh structures into the polymers.

The survey XPS spectra of P(PhPy-TPB)-Ru, P(DiPh-TPB)-Ru, and P(DiPy-TPB)-Ru (Figure S1a, Supporting Information) exhibited distinct characteristic signals assignable to O 1s, Ru 3p, N 1s, C 1s, and Cl 2p, thereby confirming the successful incorporation of O, Ru, N, C, and Cl elements in the as-prepared catalysts. In the high-resolution Ru 3p XPS spectra (Figure S1b), all three samples exhibited two relatively broad characteristic bands, which were assigned to Ru 3p3/2 and Ru 3p1/2 and located at approximately 462.6 eV and 484.7 eV, respectively. These binding energies were consistent with Ru predominantly existing in the Ru(II) oxidation state, which was in line with the expected chemical state of Ru in the NHC-coordinated complexes. ICP-OES measurements were performed to quantify the Ru loadings, and the results showed that the Ru contents in P(PhPy-TPB)-Ru, P(DiPh-TPB)-Ru, and P(DiPy-TPB)-Ru were 4.65 wt%, 4.03 wt%, and 4.63 wt%, respectively. Notably, the Ru loadings of P(PhPy-TPB)-Ru and P(DiPy-TPB)-Ru were slightly higher than that of P(DiPh-TPB)-Ru, which might be attributed to the presence of pyridine moieties that enhanced the coordination between NHC ligands and Ru species. The higher N content in P(DiPy-TPB)-Ru was consistent with the presence of two pyridine moieties per NHC ligand, confirming the successful regulation of NHC ligand structures. Furthermore, elemental (CHN) analysis was conducted to determine the nitrogen contents of the samples, which were found to be 2.18 wt%, 2.01 wt%, and 2.60 wt%, respectively.

**Figure 2:**
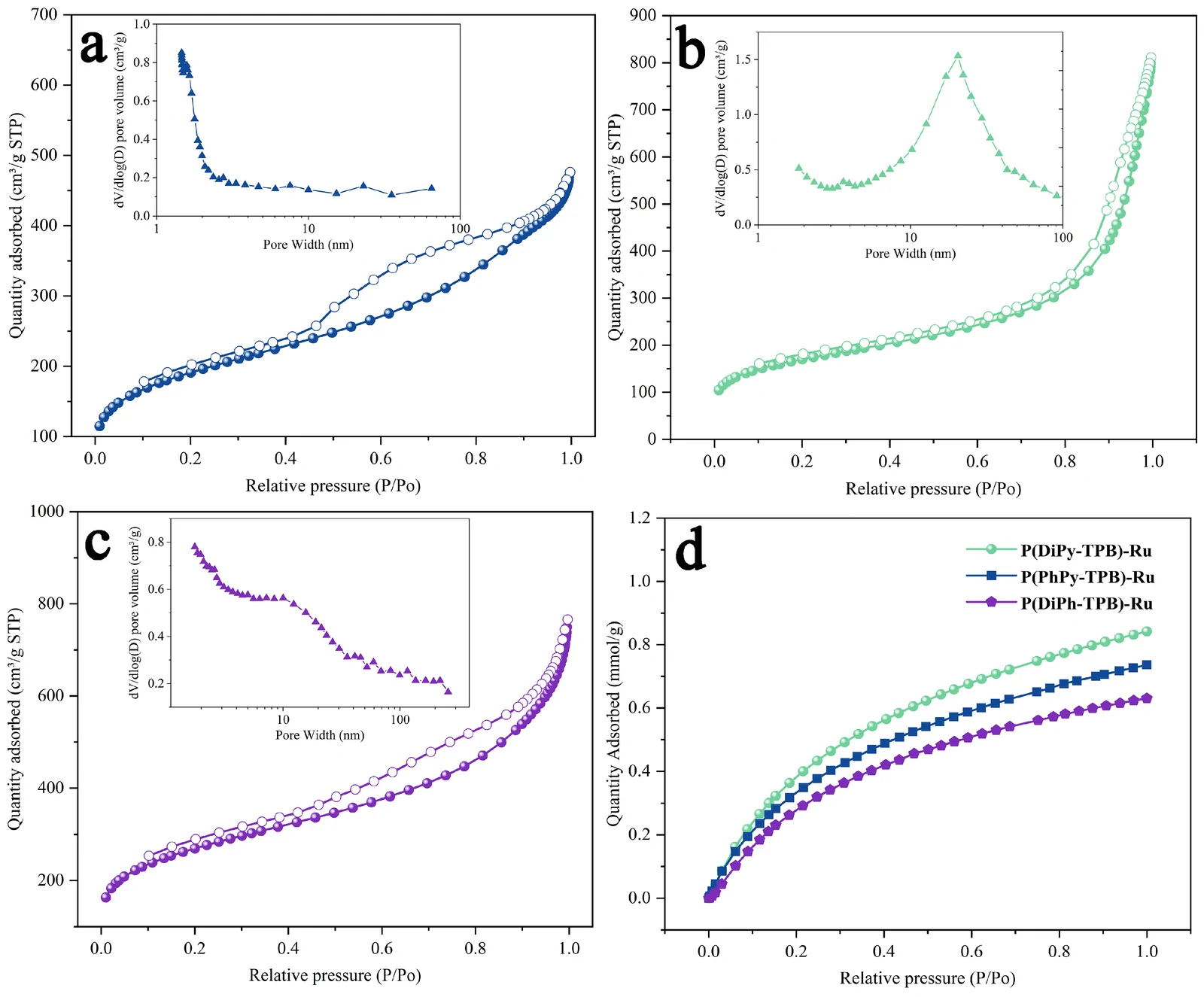
Nitrogen adsorption–desorption isotherms and pore-size distribution curves of (**a**) P(PhPy-TPB)-Ru, (**b**) P(DiPh-TPB)-Ru, and (**c**) P(DiPy-TPB)-Ru. (**d**) CO_2_ adsorption isotherms up to 1 bar at 273 K.

Nitrogen adsorption–desorption experiments were conducted at 77 K to evaluate the textural properties of the obtained materials. As summarized in Table 1, the parent hypercrosslinked polymer supports exhibited considerable porosity, Brunauer–Emmett–Teller (BET) surface areas ranging from 646 m^2^·g^−1^ to 816 m^2^·g^−1^ and total pore volumes varying from 0.54 cm^3^·g^−1^ to 1.35 cm^3^·g^−1^ (Table 1, entries 1–3). Upon the incorporation of Ru species, both textural parameters showed a modest decline, which was attributed to the partial occupation of the internal pore space by the immobilized Ru metal complexes. Accordingly, P(PhPy-TPB)-Ru, P(DiPh-TPB)-Ru, and P(DiPy-TPB)-Ru exhibited BET surface areas of 651, 580, and 721 m^2^·g^−1^, as well as pore volumes of 0.49, 1.20, and 1.14 cm^3^·g^−1^, respectively (Table 1, entries 4–6). The sorption isotherms of the Ru-containing catalysts displayed mixed type I/IV characteristics (Figure 2a–c), which indicated the presence of hierarchical porosity. The uptakes at very low relative pressures (*P*/*P*_0_ < 0.01) evidenced the presence of abundant microporosity, whereas the remarkable increase in adsorption capacity near saturation (*P*/*P*_0_ = 0.9–1.0) pointed to the existence of larger mesopores and macropores. In addition, the distinct hysteresis loops observed at high relative pressures (*P*/*P*_0_ = 0.8–1.0) further supported the contribution of mesopores. These textural features agreed well with the corresponding pore-size distributions (insets in Figure 2a–c), which revealed a broad pore-size distribution spanning the micro-, meso-, and macropore regimes. Such a combination of high BET surface area and hierarchical pore architecture is highly favorable for heterogeneous catalysis, as it can effectively enhance the accessibility of Ru active sites and accelerate the mass transfer and intraparticle diffusion of reactants and products. Figure 2d depicts the CO_2_ adsorption isotherms of the as-synthesized catalysts recorded at 273 K, which evidenced a strong affinity toward CO_2_. The measured CO_2_ uptakes were 0.74 mmol·g^−1^ for P(PhPy-TPB)-Ru, 0.63 mmol·g^−1^ for P(DiPh-TPB)-Ru, and 0.84 mmol·g^−1^ for P(DiPy-TPB)-Ru. This sequence of CO_2_ uptakes was consistent with the N content trend of the three catalysts, indicating that pyridine moieties (containing N atoms) could enhance CO_2_ adsorption.

**Figure 3:**
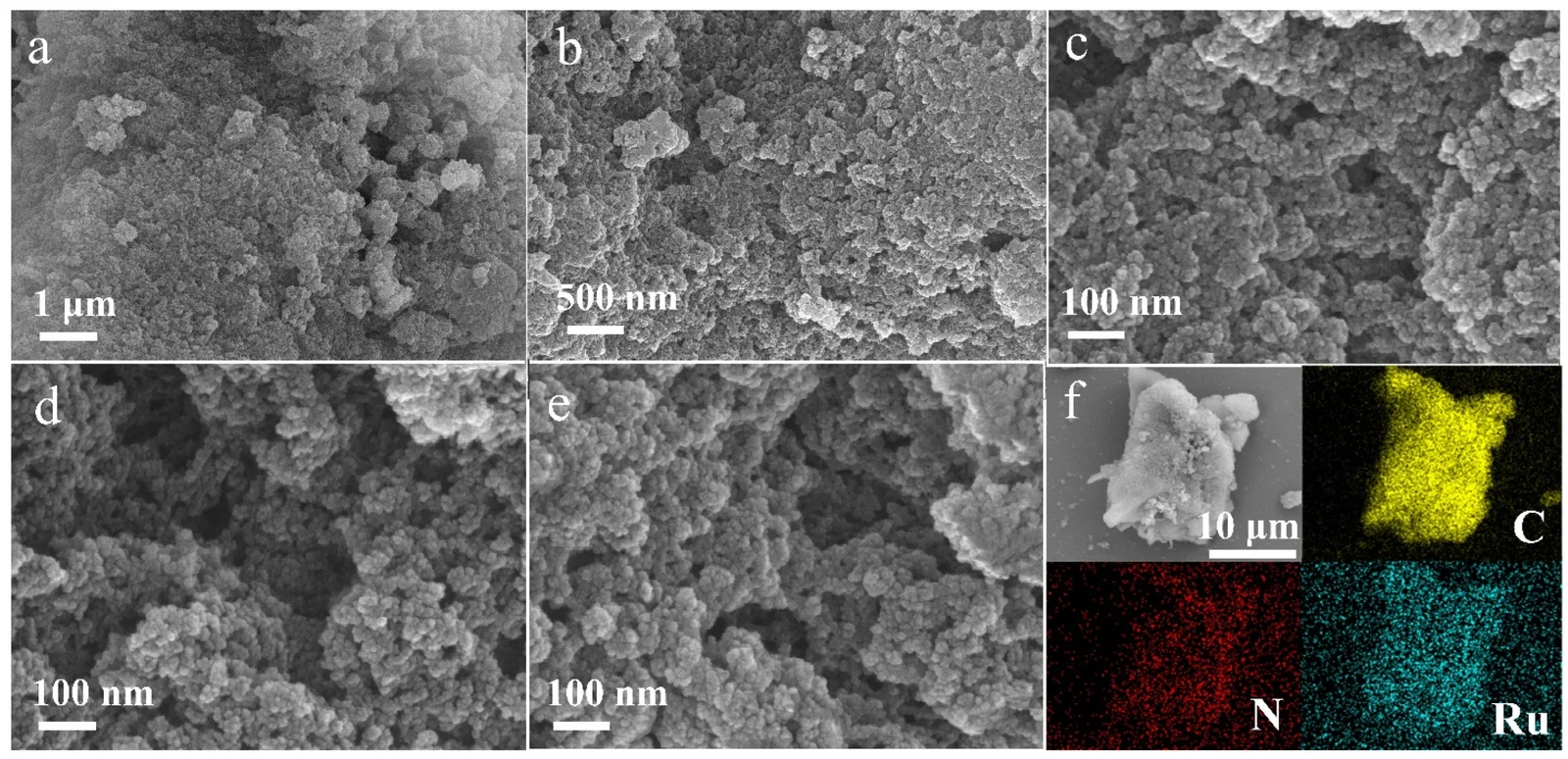
SEM images. (**a**–**c**) P(PhPy-TPB)-Ru, (**d**) P(DiPh-TPB)-Ru, (**e**) P(DiPy-TPB)-Ru, (**f**) SEM-EDS element mapping of P(PhPy-TPB)-Ru.

**Figure 4:**
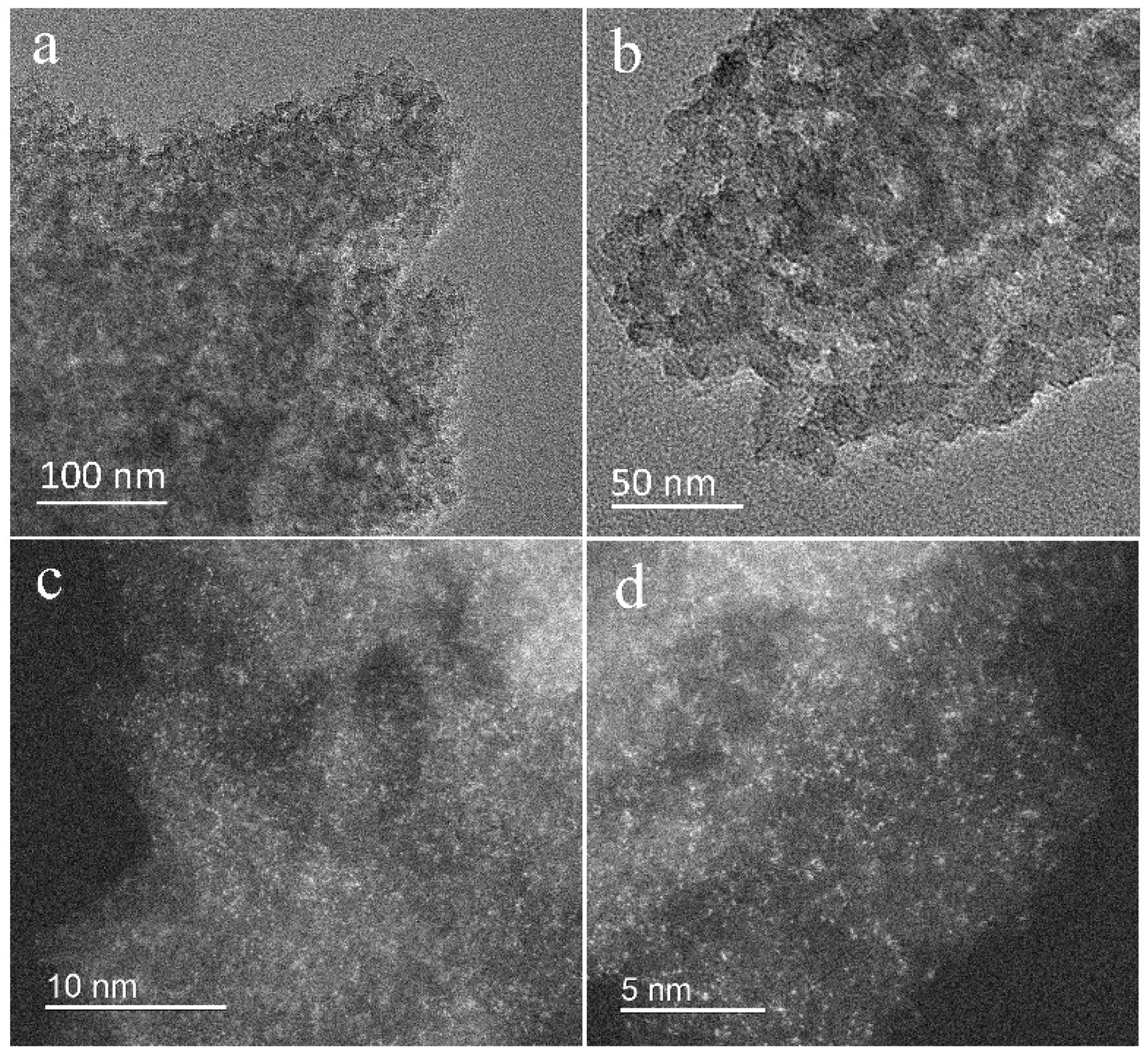
(**a**,**b**) TEM images of P(PhPy-TPB)-Ru. (**c**,**d**) AC-HAADF-STEM images of P(PhPy-TPB)-Ru.

The microstructure and morphology of the as-prepared materials were examined using SEM, SEM-EDS, TEM, and AC-HAADF-STEM. As shown in Figure 3a–e, SEM micrographs of P(PhPy-TPB)-Ru, P(DiPh-TPB)-Ru, and P(DiPy-TPB)-Ru revealed aggregates composed of tightly packed, irregular nanoparticles, with characteristic sizes in the range of several tens of nanometers. Notably, pronounced mesoporous and macroporous voids were observed between the stacked nanoparticles, which was in good agreement with the porosity trends obtained from the N_2_ adsorption–desorption measurements. Furthermore, SEM-EDS elemental mapping of P(PhPy-TPB)-Ru (Figure 3f) demonstrated a uniform distribution of C, N, and Ru elements throughout the entire sample, which indicated a uniform copolymer composition and highly dispersed Ru active centers. TEM observations (Figure 4a,b) of P(PhPy-TPB)-Ru further corroborated the compact yet porous architecture of the material and, notably, showed no discernible Ru nanoparticles, thereby implying the absence of Ru aggregation at the nanometer scale. To probe the dispersion state of Ru species at the atomic level, AC-HAADF-STEM characterization was employed. As presented in Figure 4c,d, only discrete bright spots were observed in the AC-HAADF-STEM images. These discrete bright spots are typical features of single-atom Ru species, confirming the successful fabrication of single-atom catalysts. In heterogeneous catalysis, such an atomic-level distribution of active Ru sites maximized the fraction of accessible active centers and could thereby significantly improve the overall catalytic activity and stability of the catalysts.

Thermogravimetric analysis (TGA) was performed under nitrogen atmosphere to evaluate the thermal stability of the as-prepared catalysts within the temperature range of 25–800 °C (Figure S2, Supporting Information). The three catalysts displayed nearly identical mass loss profiles, indicating similar thermal degradation behaviors. The minor weight loss observed below 100 °C was attributed to the desorption and removal of physically adsorbed guest molecules, such as trapped solvent and adsorbed water. A pronounced mass loss occurred only when the temperature exceeded approximately 220 °C, which corresponded to the thermal degradation of the polymer framework. Accordingly, the catalysts could be considered thermally stable up to at least 220 °C.

### 2.2. Catalytic N-Formylation of Amines

**Figure 5:**
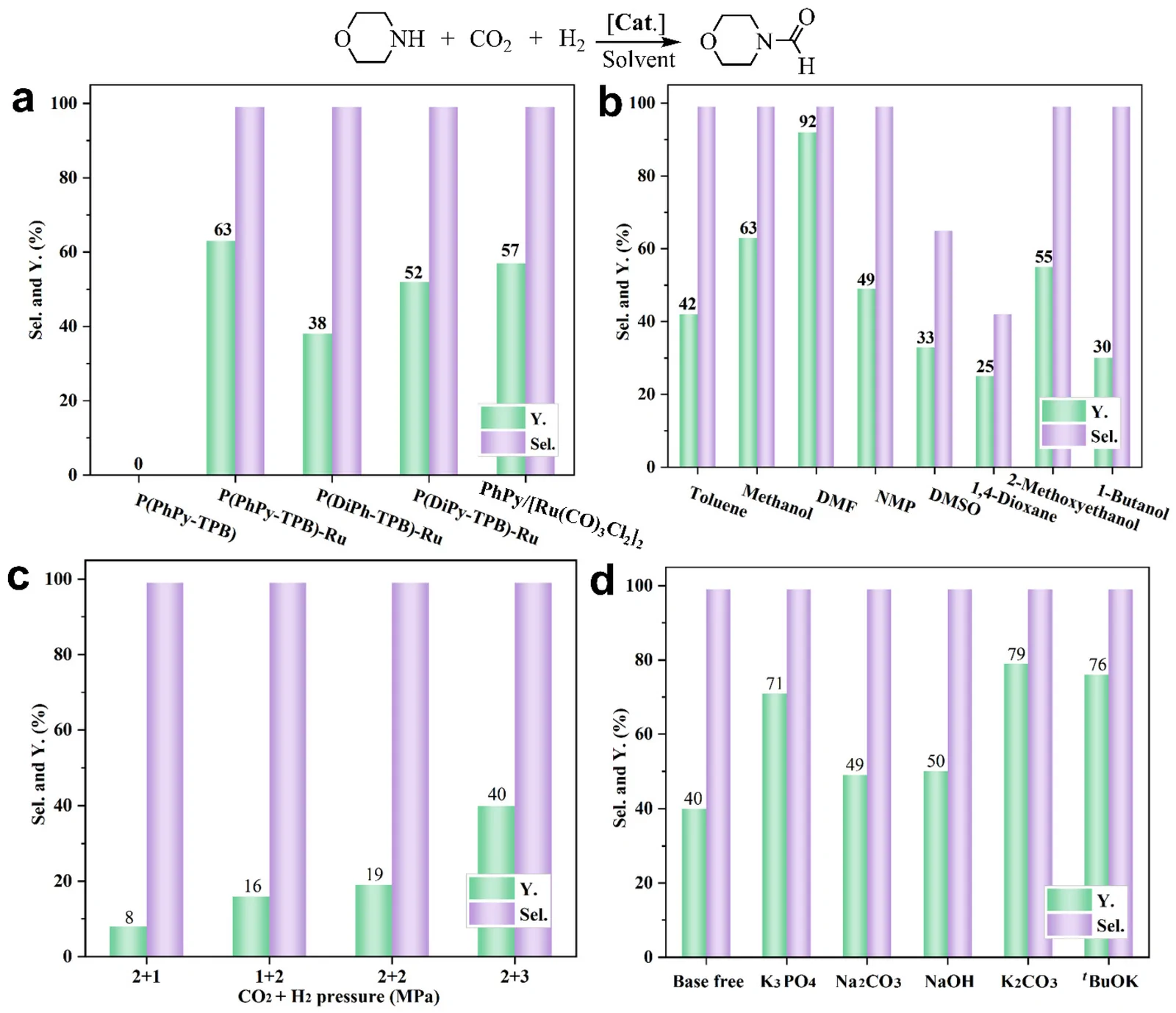
Optimization of reaction conditions in the N-formylation of morpholine. (**a**) Activity comparison of different catalysts, Ru 0.2 mol%, morpholine (3 mmol), 130 °C, 8 h, 2.0 MPa CO_2_, 3.0 MPa H_2_, 2 mL methanol. (**b**) Effect of solvent, P(PhPy-TPB)-Ru 0.2 mol%, morpholine (3 mmol), 130 °C, 8 h, 2.0 MPa CO_2_, 3.0 MPa H_2_, 2 mL solvent. (**c**) Effect of pressure, P(PhPy-TPB)-Ru 0.2 mol%, morpholine (3 mmol), 130 °C, 4 h, 1.0–2.0 MPa CO_2_, 1.0–3.0 MPa H_2_, 2 mL methanol. (**d**) Effect of base, P(PhPy-TPB)-Ru 0.2 mol%, morpholine (3 mmol), 130 °C, 4 h, 2.0 MPa CO_2_, 3.0 MPa H_2_, 2 mL methanol, base (0.2 equiv., 0.6 mmol).

The N-formylation of morpholine was chosen as a model reaction to evaluate the catalytic performance of the as-prepared samples and optimize the reaction conditions. The catalytic activities of various catalysts were first examined at 130 °C. As depicted in Figure 5a, the metal-free P(PhPy-TPB) support showed no detectable catalytic activity for N-formylmorpholine formation, confirming that Ru metal active centers are indispensable for this transformation. After immobilization of the ruthenium complex, the resulting P(PhPy-TPB)-Ru catalyst afforded N-formylmorpholine a 63% yield under identical reaction conditions. To elucidate how the NHC ligand structure affected the catalytic activity of the polymer-supported Ru catalysts, the performances of P(PhPy-TPB)-Ru, P(DiPh-TPB)-Ru, and P(DiPy-TPB)-Ru were compared under identical reaction conditions. P(DiPh-TPB)-Ru, which contained no pyridine moieties on its NHC ligand, gave only a 38% yield of N-formylmorpholine. In contrast, P(PhPy-TPB)-Ru, functionalized with one pyridine group on the NHC ligand, provided the highest yield of 63%. P(DiPy-TPB)-Ru, with two pyridine moieties on its NHC ligand, exhibited lower activity and afforded a 52% yield. Given the identical ruthenium active sites across these catalysts, the disparities in catalytic activity were ascribed to the distinct structural features of their NHC ligands. Polymeric NHC ligands incorporated with pyridine groups can enhance CO_2_ adsorption and facilitate its conversion into formic acid intermediates. However, the presence of two pyridine moieties may introduce steric hindrance and alter the electronic environment of the Ru center through coordination interactions, which ultimately reduce the catalytic activity. Notably, the heterogeneous P(PhPy-TPB)-Ru catalyst exhibited slightly higher catalytic activity than the homogeneous physical mixture of PhPy and [Ru(CO)_3_Cl_2_]_2_ (PhPy/[Ru(CO)_3_Cl_2_]_2_). After identifying P(PhPy-TPB)-Ru as the optimal catalyst, the effects of reaction parameters (solvent, pressure, base, temperature, and reaction time) were systematically investigated to maximize the catalytic performance. As shown in Figure 5b, the influence of solvent on the reaction was investigated. Among the solvents screened, DMF and methanol gave the best results, with N-formylmorpholine yields of 92% and 63%, respectively. Since DMF could act as an alternative C1 source [46], a control experiment was carried out in DMF under H_2_- and CO_2_-free conditions to assess this possibility. A comparable yield of 87% was achieved, demonstrating that the catalyst also efficiently catalyzed the N-formylation reaction when DMF served as the C1 donor. Accordingly, methanol was selected as the optimal solvent for the subsequent studies. Figure 5c illustrates the effects of CO_2_ and H_2_ pressures; to better elucidate their influence, the reaction time was shortened to 4 h. The optimal pressures were determined to be 2 MPa for CO_2_ and 3 MPa for H_2_, which favored high catalytic activity under moderate total pressure without excessive safety risks. Figure 5d presents the effect of base addition. The results demonstrated that the addition of a base significantly promoted the reaction; specifically, using 0.2 equiv. of K_2_CO_3_ as the base, a 79% yield of N-formylmorpholine was achieved after 4 h of reaction.

**Figure 6:**
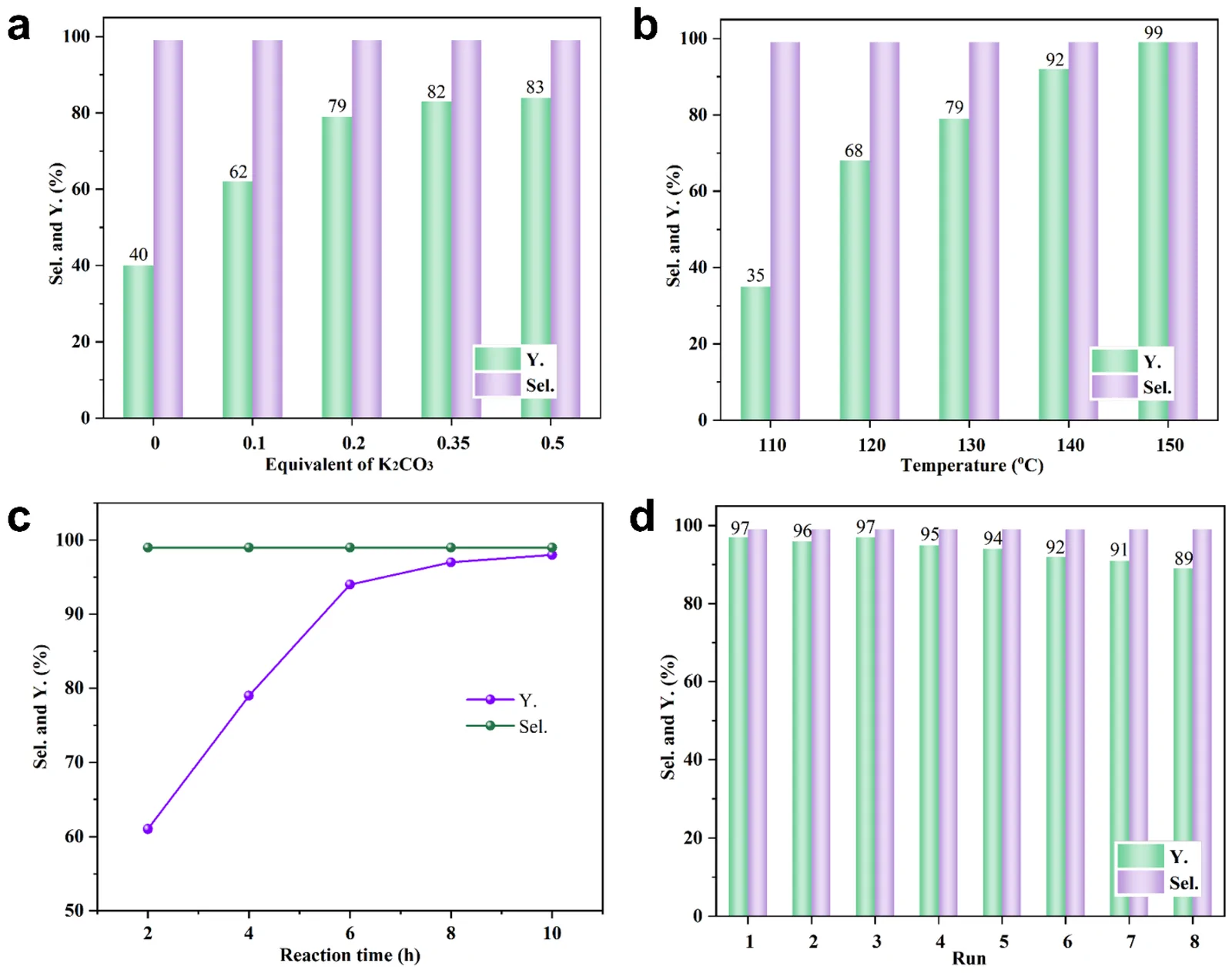
(**a**) Influence of base dosage, P(PhPy-TPB)-Ru 0.2 mol%, morpholine (3 mmol), 130 °C, 4 h, 2.0 MPa CO_2_, 3.0 MPa H_2_, 2 mL methanol, K_2_CO_3_ (0.1–0.5 equiv.). (**b**) Influence of reaction temperature, P(PhPy-TPB)-Ru 0.2 mol%, morpholine (3 mmol), 4 h, 2.0 MPa CO_2_, 3.0 MPa H_2_, 2 mL methanol, K_2_CO_3_ (0.2 equiv.). (**c**) Influence of reaction time, P(PhPy-TPB)-Ru 0.2 mol%, morpholine (3 mmol), 130 °C, 2–10 h, 2.0 MPa CO_2_, 3.0 MPa H_2_, 2 mL methanol, K_2_CO_3_ (0.2 equiv., 0.6 mmol). (**d**) Recyclability of the P(PhPy-TPB)-Ru catalyst, P(PhPy-TPB)-Ru 0.2 mol%, morpholine (3 mmol), 130 °C, 8 h, 2.0 MPa CO_2_, 3.0 MPa H_2_, 2 mL methanol, K_2_CO_3_ (0.2 equiv., 0.6 mmol).

Subsequently, the effect of K_2_CO_3_ dosage on the catalytic activity was investigated. As depicted in Figure 6a, when the K_2_CO_3_ dosage was increased from 0 to 0.1 equiv. and further to 0.2 equiv., the product yield rose sequentially from 40% to 62% and then to 79%. A further increase in K_2_CO_3_ dosage (from 0.2 to 0.5 equiv.) led to only a slight improvement in catalytic activity, which might be due to the saturation of the base-promoting effect. Figure 6b illustrates the influence of reaction temperature on the reaction activity. As the temperature was elevated from 120 °C to 150 °C, the catalytic activity increased gradually, affording a maximum yield of 99% at 150 °C. However, when the reaction temperature rose to 150 °C, the total system pressure reached approximately 8 MPa, which was close to the safety limit of the autoclave (10 MPa) and thus posed potential operational risks. To balance catalytic activity and operational safety, 130 °C was chosen as the optimal reaction temperature for subsequent experiments. The effect of reaction time on the product yield was further investigated at 130 °C, as shown in Figure 6c. After 8 h of reaction, the yield of the target product (N-formylmorpholine) reached as high as 97%, and no significant yield improvement was observed with further extension of reaction time (up to 10 h). Following the optimization of reaction conditions, the recyclability of the P(PhPy-TPB)-Ru catalyst was evaluated to assess its practical application potential. As presented in Figure 6d, the P(PhPy-TPB)-Ru catalyst exhibited excellent recyclability: over eight consecutive catalytic cycles, the yield of N-formylmorpholine remained above 89% with unchanged product selectivity (≥99%), confirming the high structural and catalytic stability of the catalyst during the reaction.

**Figure 7:**
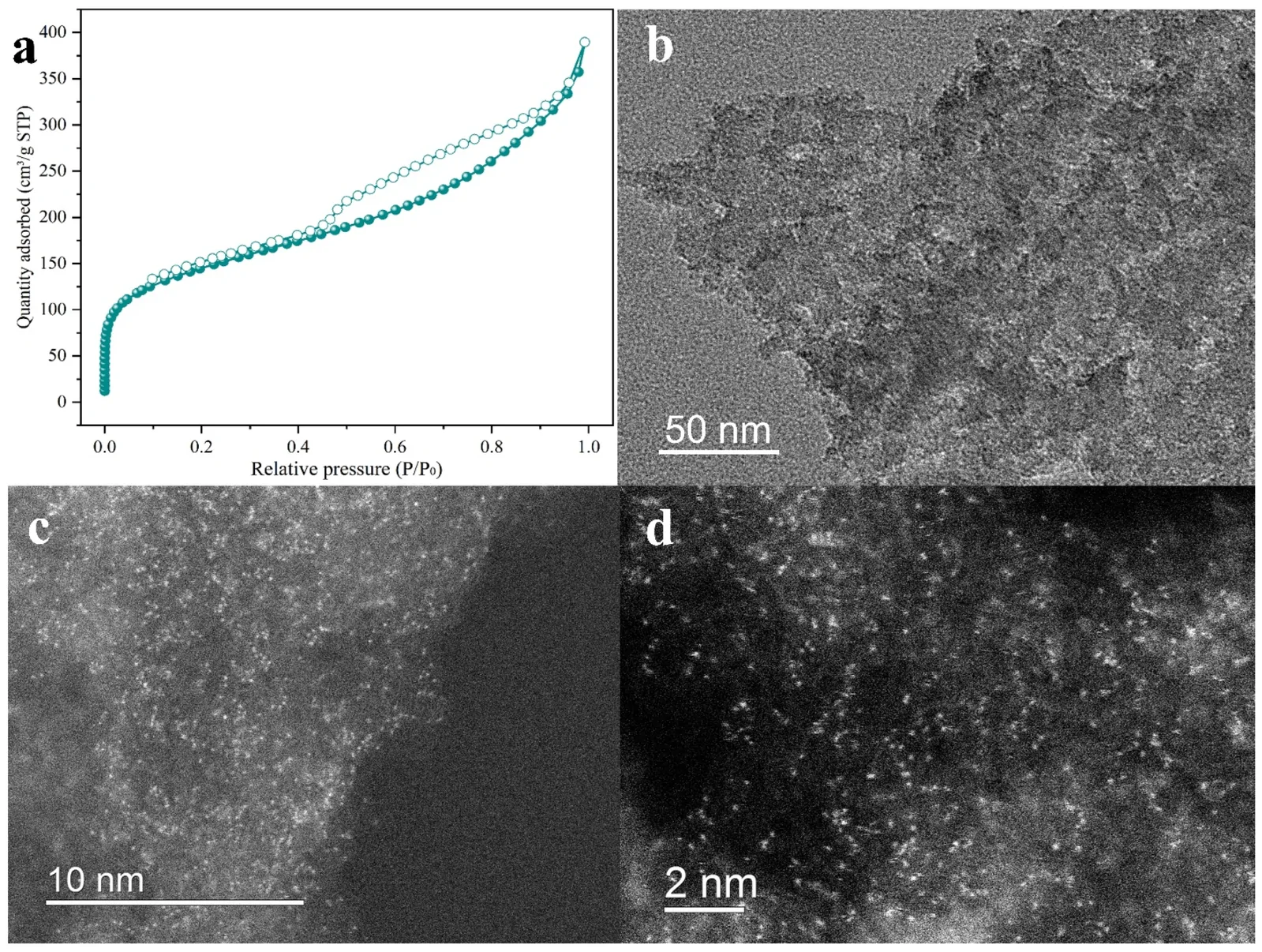
Characterization of the spent P(PhPy-TPB)-Ru catalyst after using eight times. (**a**) Nitrogen adsorption–desorption isotherms; (**b**) TEM, (**c**,**d**) AC-HAADF-STEM.

For an in-depth evaluation of the recyclability and durability of P(PhPy-TPB)-Ru, the spent catalyst (after eight cycles) was subjected to a comprehensive set of characterization techniques, including N_2_ sorption analysis, TEM, AC-HAADF-STEM, and ICP-OES. As shown in Figure 7a, the N_2_ adsorption–desorption isotherm of the spent catalyst closely resembled that of the fresh sample. The recovered P(PhPy-TPB)-Ru retained a BET specific surface area of 498 m^2^·g^−1^ and a total pore volume of 0.65 cm^3^·g^−1^ (Table 1, entry 7). The slight decreases in these textural parameters were attributed to partial pore blockage caused by residual reactants/products or minor structural rearrangement during repeated catalytic cycles. TEM analysis (Figure 7b) confirmed the absence of Ru nanoparticles in the spent catalyst, while AC-HAADF-STEM images (Figure 7c,d) further verified that Ru species remained atomically dispersed within the catalyst matrix. ICP-OES quantification results showed that the spent P(PhPy-TPB)-Ru catalyst had a Ru loading of 4.61 wt%, corresponding to a retention rate of more than 99% after eight consecutive catalytic runs. Collectively, these characterization results clearly demonstrated the excellent recycling stability and structural durability of the P(PhPy-TPB)-Ru catalyst, which is crucial for its practical application in heterogeneous catalysis.

To assess the practical applicability of P(PhPy-TPB)-Ru, its catalytic performance was evaluated for the N-formylation of various amines (cyclic, linear aliphatic, and aromatic amines) under the optimized reaction conditions. As shown in Table 2, cyclic amines, including pyrrolidine, azepane and 1,2,3,4-tetrahydroisoquinoline were efficiently converted to the corresponding formamides with high yields under the optimized reaction conditions (Table 2, entries 1–3), demonstrating the excellent adaptability of the catalyst to cyclic amine substrates. For secondary linear amines (diethylamine and dimethylamine), the target formamides were afforded 95% and 92% yields, respectively, after extending the reaction time to 12 h and 16 h. The lower reactivity of secondary linear amines compared with cyclic amines was attributed to the higher steric hindrance effects associated with their linear molecular structures (Table 2, entries 4 and 5). n-Hexylamine, a typical primary linear amine, exhibited high reactivity and afforded N-hexylformamide a 93% yield within 8 h (Table 2, entry 6). In addition, benzylamine showed lower reactivity than cyclic and linear amines; the target formamide achieved a 91% yield only when the reaction time was prolonged to 24 h (Table 2, entry 7). However, the P(PhPy-TPB)-Ru catalyst displayed poor catalytic activity for the N-formylation of aromatic amines (aniline and N-methylaniline), yielding only 57% and 43% of the corresponding formamides, respectively, even after 24 h of reaction at an elevated temperature of 140 °C. This inferior catalytic performance was presumably ascribed to the weak nucleophilicity inherent to these aromatic amines, which hindered their nucleophilic attack on the formic acid derivative intermediate during the reaction process.

### 2.3. Plausible Reaction Mechanism

**Figure 8:**
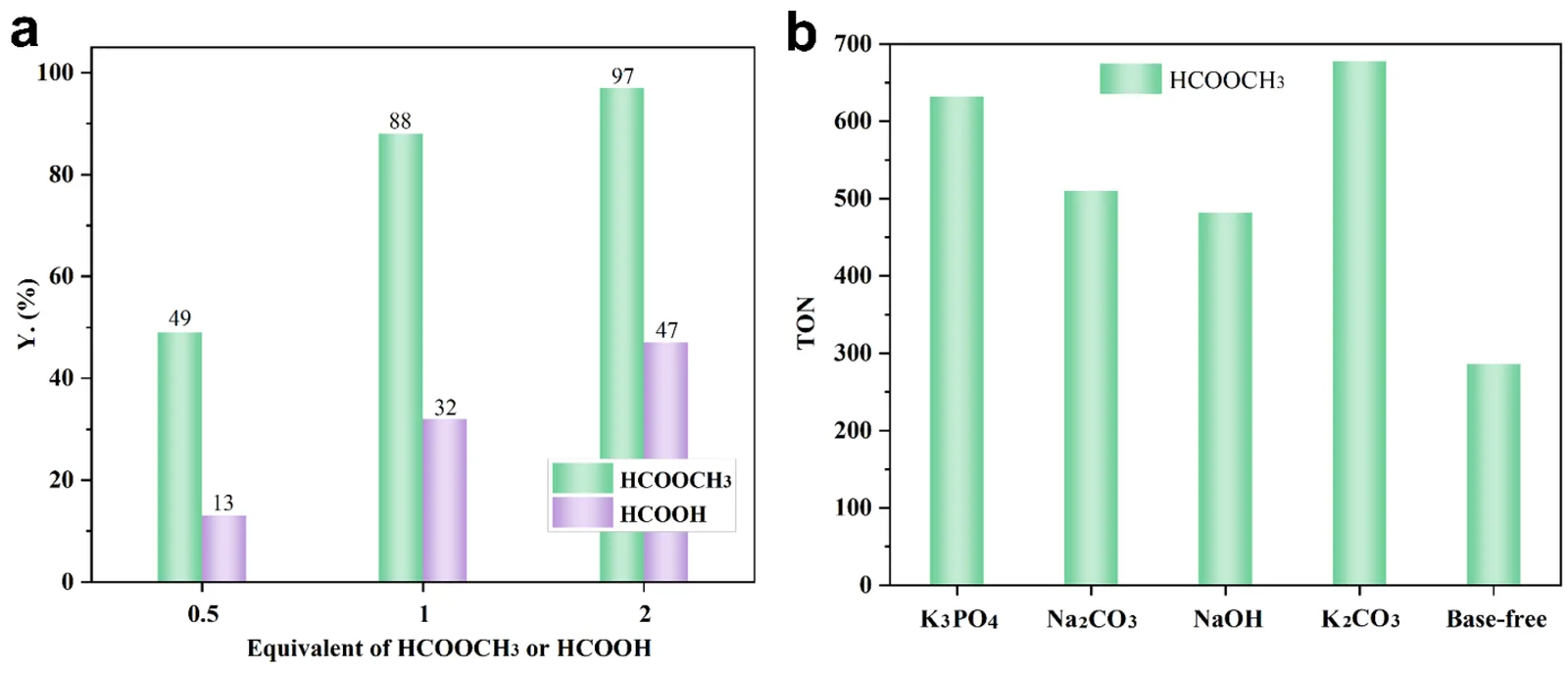
(**a**) N-formylation of morpholine using either methyl formate or formic acid as the C1 source; reaction conditions: P(PhPy-TPB)-Ru 0.2 mol%, morpholine (3 mmol), 130 °C, 8 h, 2 mL 1,4-dioxane, K_2_CO_3_ (0.6 mmol). (**b**) CO_2_ hydrogenation to methyl formate; reaction conditions: P(PhPy-TPB)-Ru 0.2 mol%, 130 °C, 4 h, 2.0 MPa CO_2_, 3.0 MPa H_2_, base (0.6 mmol), 2 mL methanol.

For the N-formylation of amines with CO_2_ and H_2_, formic acid is generally regarded as a reaction intermediate in most catalytic systems. However, in the N-formylation of morpholine with CO_2_ and H_2_ using methanol as the solvent, we detected a small amount of methyl formate, indicating that methyl formate (HCOOCH_3_) might serve as a reaction intermediate under such conditions. To gain deeper insights into the reaction mechanism, control experiments were conducted for the N-formylation of morpholine in 1,4-dioxane, using methyl formate (HCOOCH_3_) or formic acid (HCOOH) as the sole C1 source. As depicted in Figure 8a, the yield of N-formylmorpholine increased gradually with increasing molar amounts of HCOOH or HCOOCH_3_, indicating a positive correlation between the amount of C1 source and the reaction yield. Notably, at the same molar concentration of the C1 source, HCOOCH_3_ afforded a significantly higher yield of N-formylmorpholine than HCOOH. When 0.5 equivalent of HCOOCH_3_ was employed as the C1 source, the yield of N-formylmorpholine reached 49%, whereas only a 13% yield was achieved with 0.5 mmol of HCOOH. In addition, two equivalents of HCOOCH_3_ were sufficient to obtain a high yield of N-formylmorpholine within 8 h, while the yield obtained with the same molar amount of HCOOH was only 47%. These results indicated that the HCOOCH_3_-involved pathway was more efficient than the HCOOH-mediated route for the N-formylation reaction. The higher efficiency of the HCOOCH_3_-mediated pathway could be rationalized by the different reaction pathways between HCOOH and HCOOCH_3_. In the presence of HCOOH, the amine easily forms a quaternary ammonium salt with HCOOH, which reduces the nucleophilicity of the amine nitrogen center and thereby inhibits its nucleophilic attack on the carbonyl carbon of the formic acid intermediate. In contrast, in the HCOOCH_3_-mediated system, the amine retains a free lone pair on the nitrogen atom and can directly undergo nucleophilic attack on the carbonyl carbon of the HCOOCH_3_-derived intermediate, thereby efficiently yielding the target N-formylated product [29,47]. Furthermore, control experiments for CO_2_ hydrogenation were conducted in the absence of amines to verify the formation of methyl formate. As shown in Figure 8b, a significant amount of methyl formate was detected in the reaction system. The effect of the base on the formation rate of methyl formate was also investigated, and a distinct promoting effect was observed. Importantly, this promoting effect of K_2_CO_3_ on methyl formate formation was consistent with its promoting effect on the N-formylation of morpholine, suggesting that methyl formate is a probable intermediate in the reaction pathway.

**Scheme 2:**
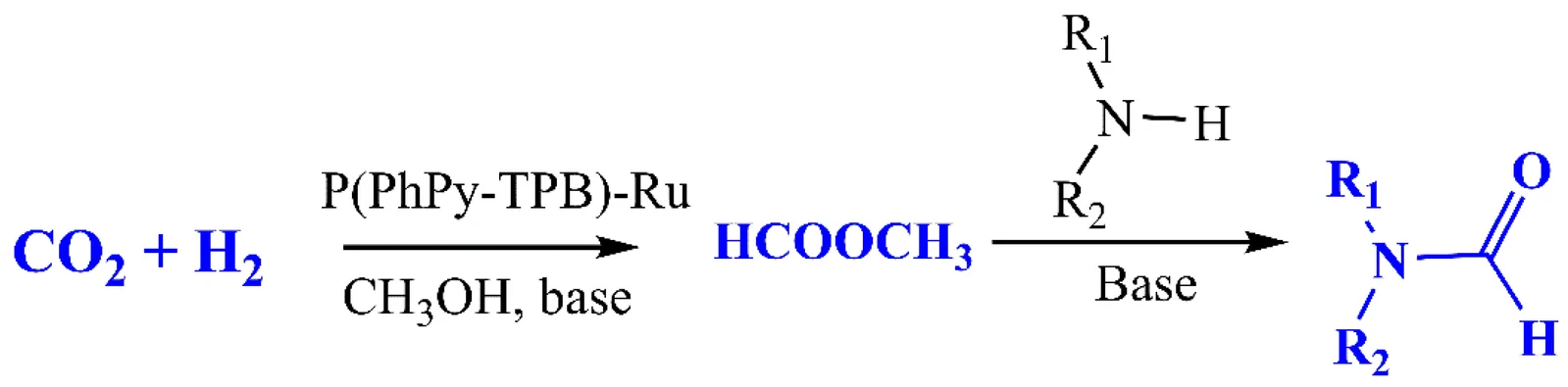
Plausible reaction path.

Based on the above control experiments and catalytic results, a plausible reaction mechanism for the P(PhPy-TPB)-Ru-catalyzed N-formylation of amines with CO_2_ and H_2_ is proposed (Scheme 2). Specifically, in the presence of methanol as the solvent and base as the promoter, CO_2_ was first hydrogenated under the catalytic action of P(PhPy-TPB)-Ru to form the methyl formate (HCOOCH_3_) intermediate, which was consistent with the earlier detection of methyl formate in the CO_2_ hydrogenation experiment without amines. Subsequently, the amine substrate, which retained a free lone electron pair on its nitrogen atom (unlike the amine-quaternary ammonium salt formation in the formic acid-mediated pathway), directly underwent nucleophilic attack on the carbonyl carbon of the HCOOCH_3_ intermediate, followed by the elimination of methanol, to ultimately yield the corresponding N-formylated product. Beyond this methyl formate-mediated route, a competing formate/formic acid-dependent pathway is also chemically feasible and widely documented in the existing literature, which may also contribute to the overall target transformation.

## 3. Materials and Methods

### 3.1. Reagents

Benzimidazole (98%), 2-(bromomethyl)pyridine hydrobromide (98%), 1-benzylbenzimidazole (95%), benzyl bromide (99.5%), sodium carbonate (99%), acetonitrile (99.9%), 1,3,5-triphenylbenzene (TPB, 98%), formaldehyde dimethyl acetal (98%, FDA), 1,2-dichloroethane (98%), anhydrous FeCl_3_ (99.9%), tricarbonyldichlororuthenium (II) dimer (95%), potassium carbonate (99%), methanol (99.5%), *N*,*N*-dimethylformamide (DMF, 99.5%), and amines were purchased from Energy Chemical Co., Ltd. (Shanghai, China). and used without further purification. *N*,*N*’-Dibenzylbenzimidazolium bromide (DiPh), N-benzyl-N’-picolylbenzimidazolium bromide (PhPy), and *N*,*N*’-dipicolylbenzimidazolium bromide (DiPy) were prepared following the procedure reported in the literature [42]; detailed experimental procedures are provided in the Supporting Information.

### 3.2. Synthesis of P(PhPy-TPB), P(DiPh-TPB), P(DiPy-TPB)

In a 48 mL pressure tube, PhPy (2 mmol), TPB (1 mmol), FDA (18 mmol), and 1,2-dichloroethane (20 mL) were added. The mixture was stirred at room temperature for 15 min. After purging with N_2_, the reaction solution was stirred at 45 °C for 6 h, and the temperature was then raised to 80 °C for an additional 18 h. After the reaction was completed, the mixture was cooled to room temperature. The resulting solid was washed three times successively with dichloromethane, dimethylformamide, water, and ethanol, and then dried under vacuum at 40 °C for 24 h to afford the polymer P(PhPy-TPB). By replacing 2 mmol of PhPy with 2 mmol of DiPh and 2 mmol of DiPy in turn, while keeping all other reaction conditions identical, P(DiPh-TPB) and P(DiPy-TPB) were synthesized following the same procedure.

### 3.3. Synthesis of P(PhPy-TPB)-Ru, P(DiPh-TPB)-Ru, P(DiPy-TPB)-Ru

In a 48 mL pressure tube, DMF (15 mL), P(PhPy-TPB) (0.5 g), [Ru(CO)_3_Cl_2_]_2_ (0.125 mmol), and triethylamine (2.5 mmol) were added. The mixture was heated and stirred in an oil bath at 110 °C for 16 h. After completion of the reaction, the mixture was cooled to room temperature. The resulting solid was washed with methanol and then dried under vacuum at 40 °C to afford the product P(PhPy-TPB)-Ru. By replacing 0.5 g of P(PhPy-TPB) with 0.5 g of P(DiPh-TPB) and 0.5 g of P(DiPy-TPB), respectively, while keeping all other reaction conditions unchanged, P(DiPh-TPB)-Ru and P(DiPy-TPB)-Ru were prepared separately.

### 3.4. N-Formylation of Amines with CO_2_ and H_2_

In a 25 mL high-pressure stainless-steel autoclave, amine (3 mmol), Ru catalyst, and solvent (3 mL) were added, and the autoclave was subsequently sealed. The air inside the autoclave was thoroughly purged with CO_2_, after which the autoclave was pressurized with predetermined pressures of CO_2_ and H_2_. The reaction was then carried out under the predetermined reaction conditions for 4–24 h. After the reaction was completed, the autoclave was cooled to room temperature. The resulting mixture was separated via high-speed centrifugation. Selectivity and yields were determined by gas chromatography (GC). For recycling experiments, the solid catalyst was recovered by centrifugation after each catalytic run, washed three times with methanol, centrifuged at 6000 rpm for 5 min, dried under vacuum at 40 °C for 24 h, and then directly reused for the subsequent catalytic reactions.

### 3.5. Characterization

The synthesized materials were subjected to a comprehensive array of physicochemical characterizations, including Fourier transform infrared (FTIR) spectroscopy, solid-state ^13^C CP-MAS NMR, X-ray photoelectron spectroscopy (XPS), elemental analysis, inductively coupled plasma-optical emission spectrometry (ICP-OES), scanning electron microscopy (SEM), transmission electron microscopy (TEM), aberration-corrected high-angle annular dark-field scanning transmission electron microscopy (AC-HAADF-STEM), N_2_ adsorption–desorption analysis, CO_2_ adsorption analysis, and thermogravimetric analysis. The detailed methodologies of these characterization techniques are available in the Supplemental Files.

## 4. Conclusions

In summary, a series of single-atom ruthenium (Ru) catalysts supported on pyridine-modified NHC-functionalized hypercrosslinked polymers, featuring high surface areas, hierarchical pore structures, and single-atom Ru dispersion, were successfully prepared. The number of pyridine moieties on the NHC ligand had a significant effect on the CO_2_ adsorption capacity and catalytic performance of the catalysts. Specifically, P(PhPy-TPB)-Ru, functionalized with a single pyridine moiety appended to the NHC ligand, emerged as the optimal catalyst in the N-formylation of amines with CO_2_ and H_2_. Furthermore, a plausible reaction mechanism involving methyl formate as the intermediate is proposed. This heterogeneous catalyst exhibited excellent reusability, retaining high catalytic activity even after eight consecutive reaction cycles. Additionally, several primary and secondary aliphatic amines were efficiently converted into the corresponding formamide products with high yields. This study not only offers an active and reusable solid catalyst for the N-formylation of amines but also furnishes some theoretical and experimental insights for the rational design of high-performance solid catalysts in CO_2_ hydrogenation reactions.

## Figures and Tables

**Table 1:** Textural properties of the prepared catalysts.

Entry	Samples	*S*_BET_ ^1^ (m^2^/g)	*V*_p_ ^2^ (m^3^/g)	*P* (nm)
1	P(PhPy-TPB)	704	0.54	5.44
2	P(DiPh-TPB)	646	1.24	9.57
3	P(DiPy-TPB)	816	1.35	5.29
4	P(PhPy-TPB)-Ru	651	0.49	4.69
5	P(DiPh-TPB)-Ru	580	1.20	8.48
6	P(DiPy-TPB)-Ru	721	1.14	5.47
7 ^3^	P(PhPy-TPB)-Ru	498	0.52	4.76

^1^ BET surface area. ^2^ Single point adsorption total pore volume of pores at *P*/*P*_0_ = 0.95. ^3^ The recovered P(PhPy-TPB)-Ru after using 8 times.

**Table 2:** N-formylation of other amines using CO_2_ and H_2_ ^1^.

Entry	Substrate	Product	Reaction Time (h)	Yield (%) ^2^
1	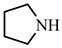	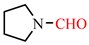	8	98
2	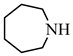	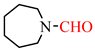	8	99
3	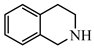	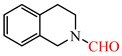	8	96
4	** 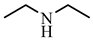 **	** 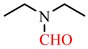 **	12	95
5 ^3^	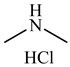	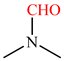	16	92
6	** 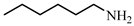 **	** 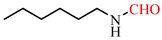 **	8	93
7	** 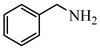 **	** 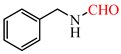 **	24	91
8 ^4^	** 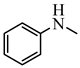 **	** 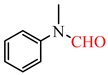 **	24	57
9 ^4^	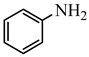	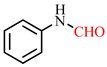	24	43

^1^ Reaction conditions: amine (3 mmol), P(PhPy-TPB)-Ru (0.2 mol%), CH_3_OH (2 mL), P_H2_ (3 MPa), P_CO2_ (2 MPa), 130 °C, K_2_CO_3_ (0.6 mmol). ^2^ Determined by GC. ^3^ K_2_CO_3_ (3.6 mmol). ^4^ 140 °C.

## Data Availability

The data presented in this study are available on request from the corresponding authors.

## Supplementary Materials

The following supporting information can be downloaded at: https://www.mdpi.com/article/10.3390/molecules31173054/s1, Section S1. Experiment and Characterization; Section S2. Characterization Results; Section S3. NMR Data. Refs. [48,49] are cited in the Supplementary Materials. Figure S1: XPS analysis. (a) Full spectra, (b) Ru 3p. Figure S2: TGA curves of the prepared catalysts.
