# Supplementary material for: Single-Atom Ru on N-Heterocyclic Carbene-Functionalized Hypercrosslinked Polymers for N-Formylation of Amines with CO2 and H2
Source: Molecules. 2026 Aug 31;31(17):3054. doi: 10.3390/molecules31173054 (PMC13567192; doi:10.3390/molecules31173054)

Supporting Information

Single-atom Ru on N-Heterocyclic Carbene-functionalized Hypercrosslinked Polymers for N-Formylation of Amines with CO_2 and H_2

Lulu Dang [1,2], Yinfang Gao [1,2], Ya-Li Wan[*,3], Qingsong Li [4], Yizhu Lei [1,2,*]

[1] *College of Environmental and Chemical Engineering, Dalian University, Dalian 116622, PR China; 468725439@qq.com (L.D.); 2594753788@qq.com (Y.G.); yzleiabc@lpssy.edu.cn (Y.L.)*

[2] *Guizhou Provincial Key Laboratory of Green Catalysis and Materials for resource conversion, School of Chemistry and Materials Engineering, Liupanshui Normal University, Liupanshui, Guizhou, 553004, PR China; 468725439@qq.com (L.D.); 2594753788@qq.com (Y.G.); yzleiabc@lpssy.edu.cn (Y.L.)*

[3] *School of Chemical Engineering, Guizhou Institute of Technology, Guiyang 550025, PR China; yaliwan06@163.com (Y.W.)*

[4] *Guizhou Laboratory of Energy Intelligent Development and Efficient Utilization, Guizhou Research Institute of Coal Mine Design Co., Ltd., Guiyang 550025, PR China; 13608615@qq.com (Q.L.)*

* *Correspondence: yzleiabc@lpssy.edu.cn (Y.L.); yaliwan06@163.com (Y.W.)*

Section S1. Experiment and Characterization

1.1 Synthesis of *N,N′*-Dibenzylbenzimidazolium bromide (DiPh) [48]

N,N′-Dibenzylbenzimidazolium bromide (DiPh) was synthesized using the method reported in the literature with some modifications [1]. Benzimidazole (5 mmol), benzyl bromide (13 mmol), and sodium carbonate (26 mmol) were added to acetonitrile (300 mL). The reaction mixture was refluxed at 80 °C for 24 h under a nitrogen atmosphere. After the reaction finished, the solvent was removed by rotary evaporation at 35 °C. The oily residue was dissolved in dichloromethane, and the solid was removed by filtration. Subsequently, the remaining solution was concentrated by rotary evaporation. The obtained oil was washed with tetrahydrofuran (THF) three times, and the resulting solid was separated by centrifugation. The solid was dried under vacuum at 60 °C for 24 h to afford 1.44 g of *N,N′*-Dibenzylbenzimidazolium bromide (DiPh) as a white powder, with an isolated yield of 76%.

1.2 Synthesis of N-benzyl-N'-picolylbenzimidazolium bromide (PhPy)

N-benzyl-N'-picolylbenzimidazolium bromide (PhPy) was synthesized using the method reported in the literature with some modifications [49]. *N,N′*-Dibenzylbenzimidazolium bromide (DiPh) was synthesized using the method reported in the literature with some modifications 1-Benzylbenzimidazole (5 mmol), 2-(bromomethyl)pyridine hydrobromide (5.3 mmol), and sodium carbonate (10.6 mmol) were added to acetonitrile (300 mL). The reaction mixture was refluxed at 80 °C for 24 h under a nitrogen atmosphere. After the reaction finished, the solvent was removed by rotary evaporation at 35 °C. The oily residue was dissolved in dichloromethane, and the solid was removed by filtration. Subsequently, the remaining solution was concentrated by rotary evaporation. The obtained oil was washed with

tetrahydrofuran (THF) three times, and the resulting solid was separated by centrifugation. The solid was dried under vacuum at 60 °C for 24 h to afford 1.04 g of N-benzyl-N'-picolylbenzimidazolium bromide (PhPy) as a light yellow powder, with an isolated yield of 55%.

1.3 Synthesis of *N,N′*-dipicolylbenzimidazolium bromide (DiPy)

N,N'-dipicolylbenzimidazolium bromide (DiPy) was synthesized using the method reported in the literature with some modifications [42]. Benzimidazole (5 mmol), 2-(bromomethyl)pyridine hydrobromide (12 mmol), and sodium carbonate (24 mmol) were added to acetonitrile (300 mL). The reaction mixture was refluxed at 80 °C for 48 h under a nitrogen atmosphere. After the reaction finished, the solvent was removed by rotary evaporation at 35 °C. The oily residue was dissolved in dichloromethane, and the solid was removed by filtration. Subsequently, the remaining solution was concentrated by rotary evaporation. The obtained oil was washed with tetrahydrofuran (THF) three times, and the resulting solid was separated by centrifugation. The solid was dried under vacuum at 60 °C for 24 h to afford 1.52 g of *N,N′*-dipicolylbenzimidazolium bromide (DiPy) as a reddish-brown powder, with an isolated yield of 79%.

1.4 Characterization

Fourier-transform infrared spectroscopy (FTIR) was recorded on a Bruker Equinox 55 FTIR spectrophotometer (Ettlingen, Germany) in a wavenumber range of 4000-400 cm^{-1}. Solid-state ^{13}C CP-MAS NMR spectra were recorded on a Bruker AVANCE III 600 spectrometer (Ettlingen, Germany) equipped with a 4 mm MAS probe. The cross-polarization (CP) contact time was 2 ms, MAS spinning rate was 10 kHz. X-ray photoelectron spectroscopy (XPS) was recorded on a VG multilab 2000 X-ray photoelectron spectrometer (Runcorn, USA) using a Mg-$Al_{K\alpha}$ X-ray source. N elemental analyses were carried out on a Vario Micro cube Elemental Analyser (Hanau, Germany). N_2 adsorption-desorption isotherms were performed on a Micrometrics ASAP

2020 (Georgia, USA) at 77 K. Before the measurements, the samples were vacuum dried at 90 °C for 12 h. Pore size distribution of the sample was calculated by the nonlocal density functional theory (NLDFT) method. The CO_2 sorption performance was estimated in the single-composition gas on a Micrometrics ASAP 2020 (Georgia, USA) at 273 K. Scanning electron microscope (SEM) and SEM-EDS element mapping images were collected on a ZEISS GeminiSEM 300 instrument (Oberkochen, Germany). Transmission electron microscopy (TEM) was measured on a 200 kV JEM-2100 instrument (Tokyo, Japan). Thermogravimetric analysis (TGA) was recorded on a NETZSCH STA 449C (Selb, Germany) at the heating rate of 10 °C/min under nitrogen atmosphere. ^{1}H NMR and ^{13}C NMR spectra were recorded on a Bruker AVANCE III NMR spectrometer (Ettlingen, Germany) at ambient temperature. Gas chromatography (GC) was carried out on a Scientific™ TRACE™ 1310 (Massachusetts, USA) equipped with a TRACE TR-5MS capillary column and an FID detector.

Selectivity and yield of the target product were calculated by the following equations:

$$\text{Selectivity (mol\%)} = \frac{\text{moles of amide generated}}{\text{moles of amine converted}} \times 100\%$$

$$\text{Yield (mol\%)} = \frac{\text{moles of amide generated}}{\text{initial moles of amine}} \times 100\%$$

Section S2. Characterization Results

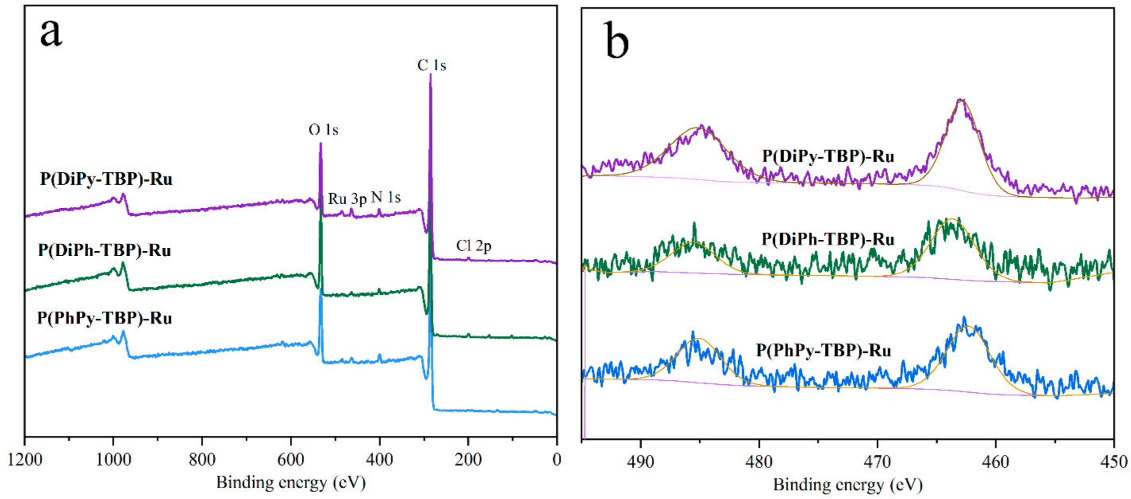

Figure S1. XPS analysis. (a) Full spectra, (b) Ru 3p.

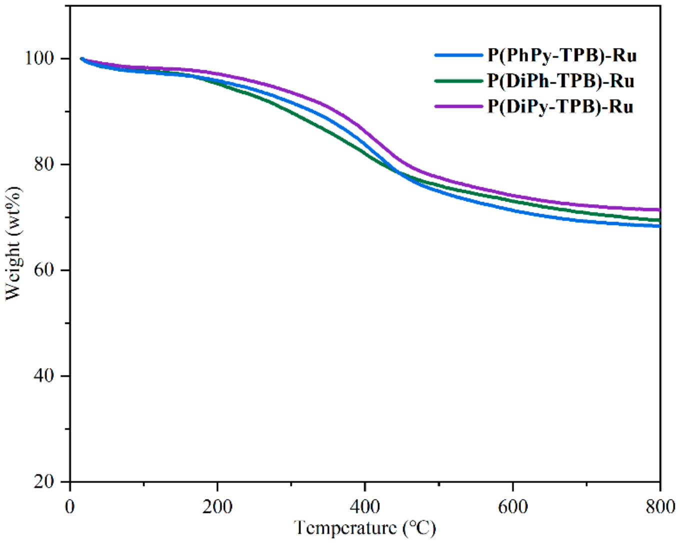

Figure S2. TGA curves of the prepared catalysts.

Section S3. NMR Data

***N,N'*-Dibenzylbenzimidazolium bromide (DiPh),** ^{1}HNMR (400 MHz, DMSO-*d*$_6$) δ = 10.27 (s, 1H, NCHN-H), 8.05 – 7.96 (m, 2H, Ar-H), 7.66 – 7.63 (dq, *J* = 6.6, 3.5 Hz, 2H, Ar-H), 7.61 – 7.53 (m, 4H, Ar-H), 7.48 – 7.34 (m, 6H, Ar-H), 5.85 (s, 4H, 2CH$_2$) ppm; ^{13}CNMR (101 MHz, DMSO-*d*$_6$) δ = 143.2, 134.5, 131.5, 129.5, 129.2, 128.8, 127.3, 114.6, 50.5 ppm.

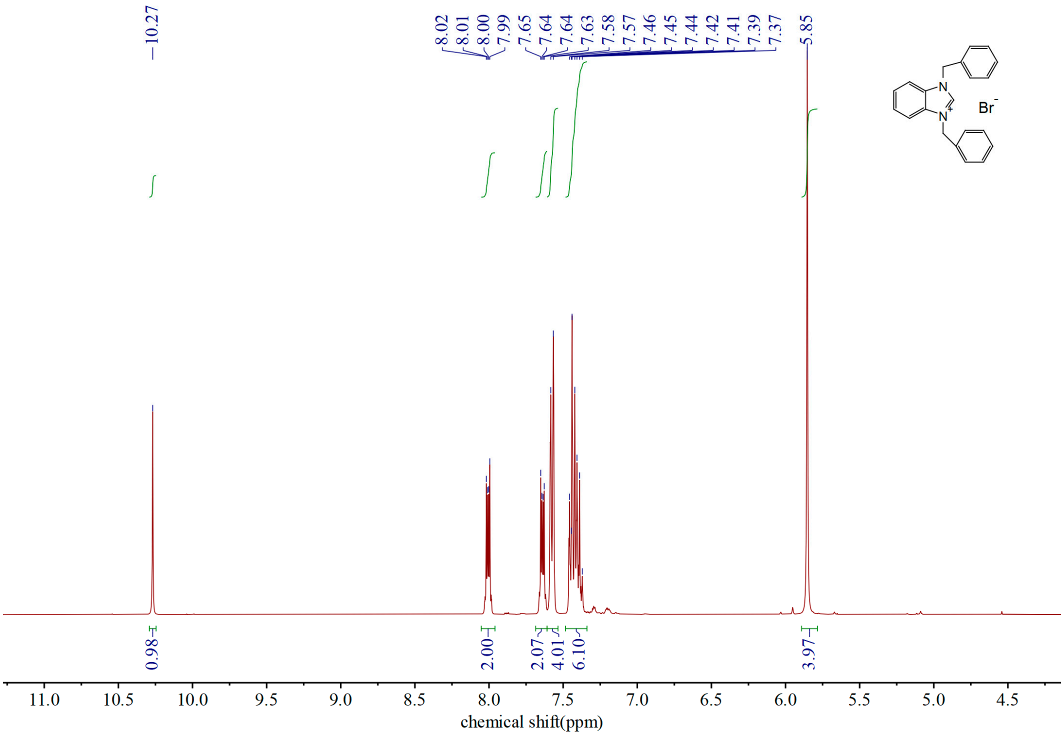

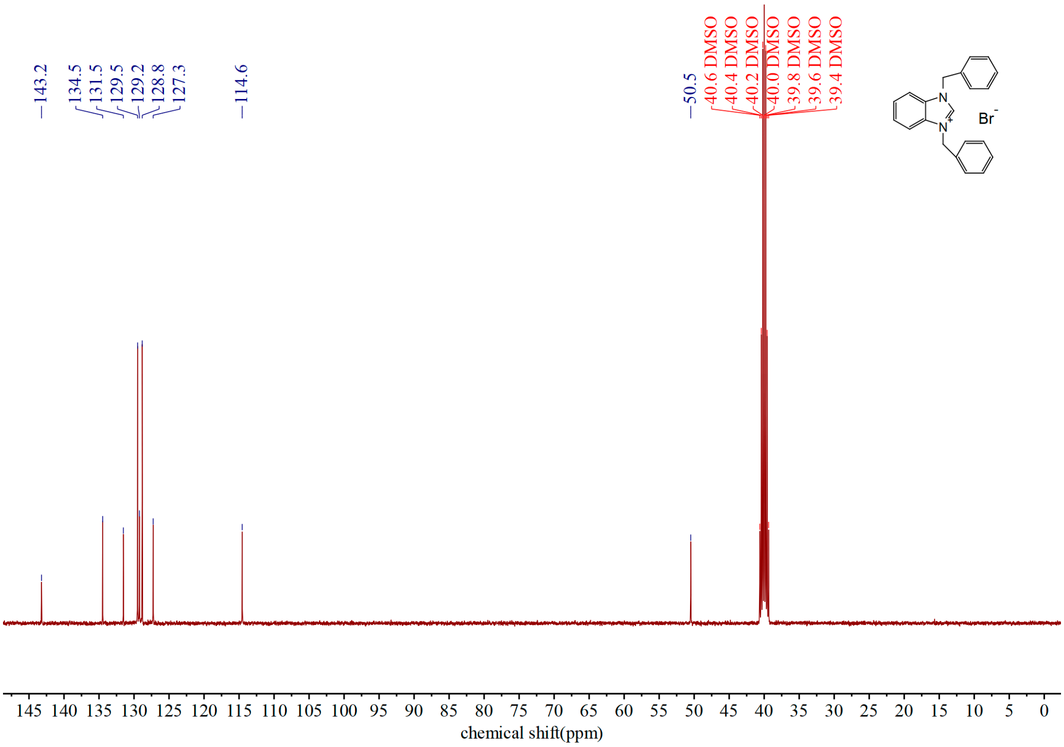

N-benzyl-N'-picolylbenzimidazolium bromide (PhPy), ^{1}HNMR (400 MHz, DMSO-d_6) δ = 10.23 (s, 1H, NCHN), 8.50 (dd, J = 5.3, 1.9 Hz, 1H，py-α-H), 8.05 – 7.88 (m, 3H), 7.72 (d, J = 7.8 Hz, 1H), 7.67 – 7.60 (m, 2H), 7.55 (d, J = 7.7 Hz, 2H), 7.46 – 7.39 (m, 4H), 6.00 (s, 2H, N-CH2), 5.89 (d, J = 2.8 Hz, 2H, N-CH2) ppm; ^{13}C NMR (101 MHz, DMSO-d_6) δ = 153.4, 150.1, 144.0, 138.0, 134.5, 132.0, 131.3, 129.5, 129.2, 128.7, 127.3, 127.2, 124.2, 123.3, 114.5, 51.4, 50.4 ppm.

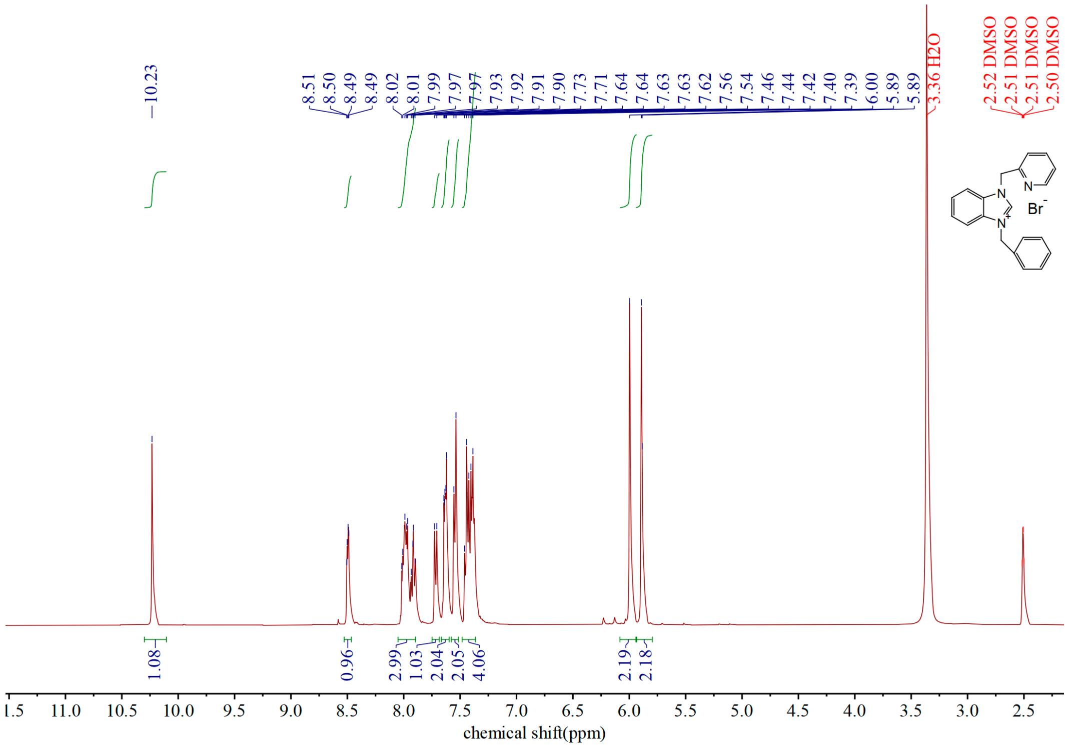

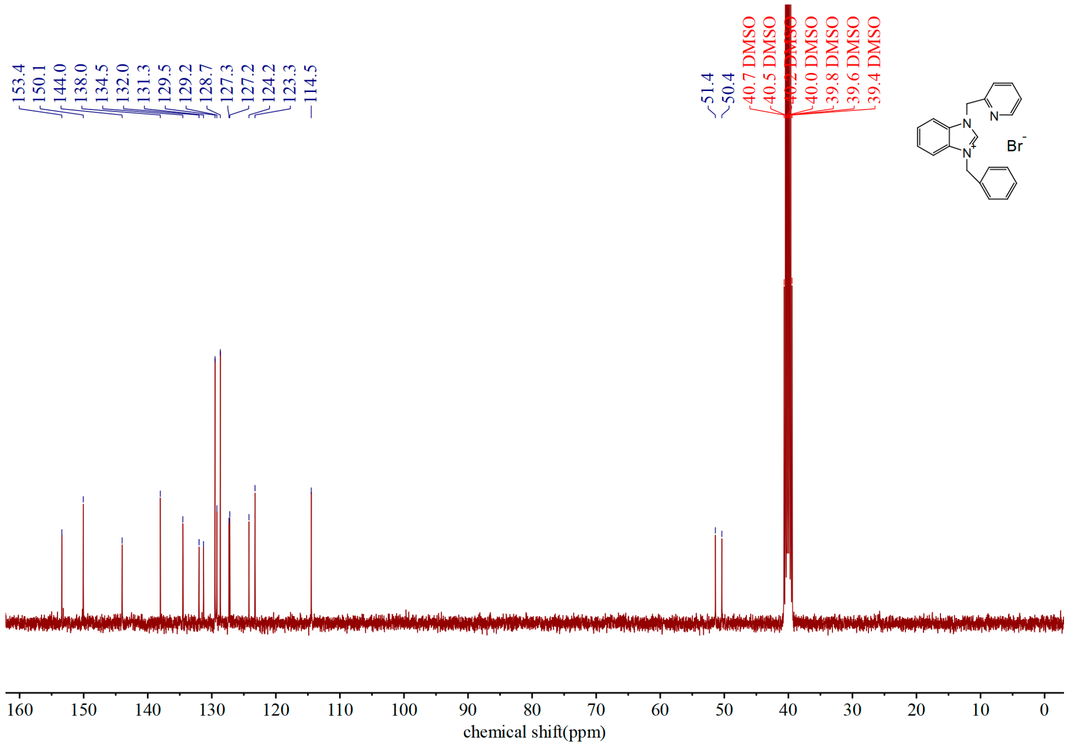

***N,N'*-dipicolylbenzimidazolium bromide (DiPy)**, ^{1}HNMR (400 MHz, DMSO-d_6) δ = 10.23 (s, 1H,NCHN), 8.51 (ddd, *J* = 4.9, 1.8, 0.9 Hz, 2H,pyridine-H), 8.04 – 7.88 (m, 4H,Ar-H), 7.72 (d, *J* = 7.8 Hz, 2H,Ar-H), 7.63 (dd, *J* = 6.3, 3.2 Hz, 2H,Ar-H), 7.44 – 7.36 (m, 2H,pyridine-H), 6.06 (s, 4H,NCH$_2$-pyridine) ppm; ^{13}C NMR (100 MHz, DMSO-d_6) δ = 153.5, 150.1, 144.4, 138.1, 131.7, 127.2, 124.2, 123.2, 114.5, 51.4 ppm.

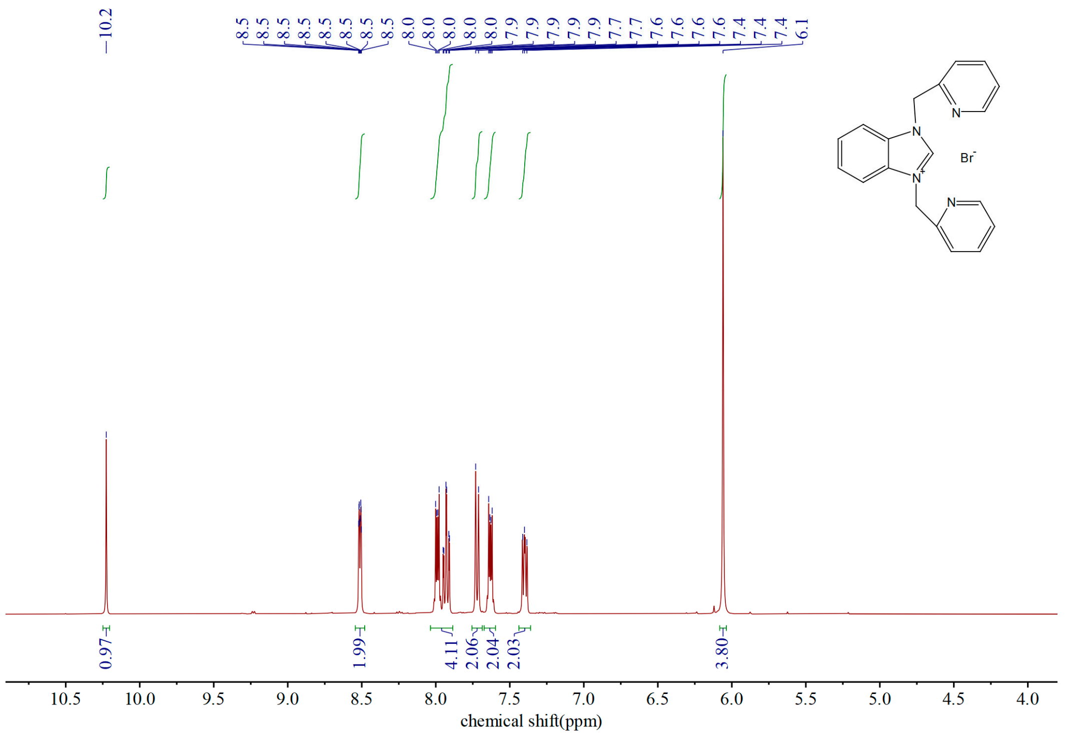

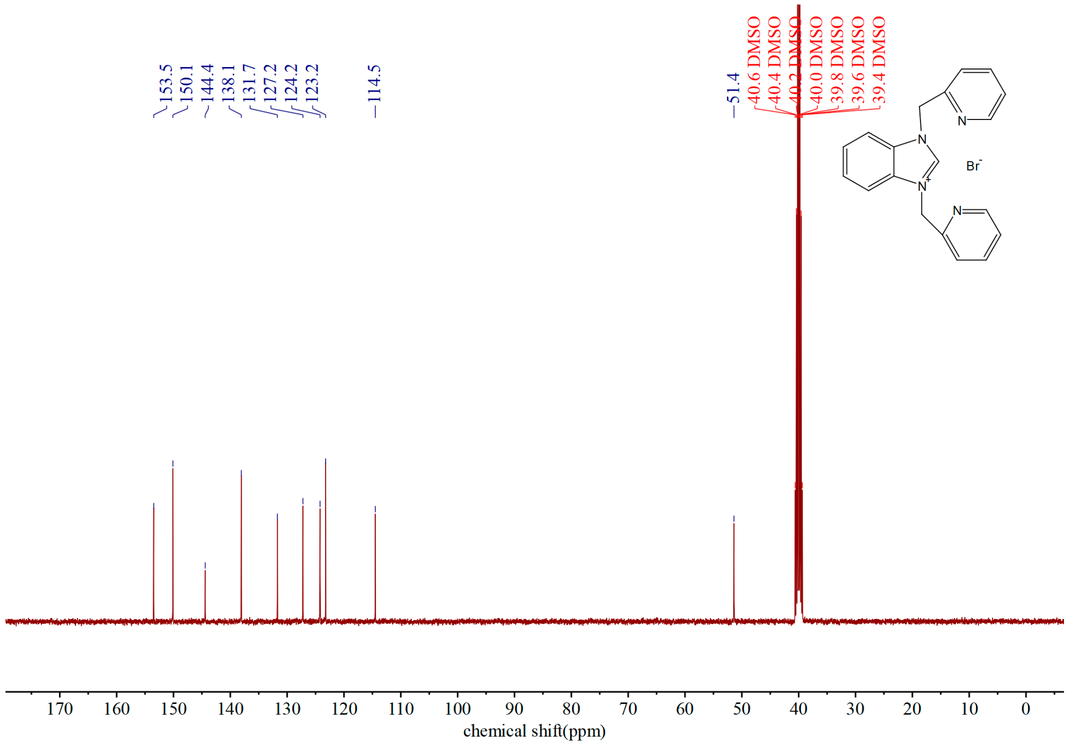

Supplement: Supplementary file 1 [file molecules-31-03054-s001.zip › molecules-4337915-supplementary.pdf]
